# Long-Term Weight Management Using Wearable Technology in Overweight and Obese Adults: Systematic Review

**DOI:** 10.2196/13461

**Published:** 2020-03-10

**Authors:** Emily Fawcett, Michelle Helena Van Velthoven, Edward Meinert

**Affiliations:** 1 Department of Paediatrics University of Oxford Oxford United Kingdom; 2 Department of Primary Care and Public Health Imperial College London London United Kingdom

**Keywords:** telemedicine, mHealth, eHealth, mobile health, obesity, wearable electronic devices, wearable technology, wearable device, digital technology, weight loss, overweight, fitness trackers

## Abstract

**Background:**

Although there are many wearable devices available to help people lose weight and decrease the rising prevalence of obesity, the effectiveness of these devices in long-term weight management has not been established.

**Objective:**

This study aimed to systematically review the literature on using wearable technology for long-term weight loss in overweight and obese adults.

**Methods:**

We searched the following databases: Medical Literature Analysis and Retrieval System Online, EMBASE, Compendex, ScienceDirect, Cochrane Central, and Scopus. The inclusion criteria were studies that took measurements for a period of ≥1 year (long-term) and had adult participants with a BMI >24. A total of 2 reviewers screened titles and abstracts and assessed the selected full-text papers for eligibility. The risk of bias assessment was performed using the following tools appropriate for different study types: the Cochrane risk of bias tool, Risk Of Bias In Nonrandomized Studies-of Interventions, A MeaSurement Tool to Assess systematic Reviews, and 6 questions to trigger critical thinking. The results of the studies have been provided in a narrative summary.

**Results:**

We included five intervention studies: four randomized controlled trials and one nonrandomized study. In addition, we used insights from six systematic reviews, four commentary papers, and a dissertation. The interventions delivered by wearable devices did not show a benefit over comparator interventions, but overweight and obese participants still lost weight over time. The included intervention studies were likely to suffer from bias. Significant variances in objectives, methods, and results of included studies prevented meta-analysis.

**Conclusions:**

This review showed some evidence that wearable devices can improve long-term physical activity and weight loss outcomes, but there was not enough evidence to show a benefit over the comparator methods. A major issue is the challenge of separating the effect of decreasing use of wearable devices over time from the effect of the wearable devices on the outcomes. Consistency in study methods is needed in future long-term studies on the use of wearable devices for weight loss.

**Trial Registration:**

PROSPERO CRD42018096932; https://www.crd.york.ac.uk/prospero/display_record.php?RecordID=96932

## Introduction

Obesity is a rising concern worldwide [1]. By 2030, obesity prevalence in the United States is predicted to be 50% to 51% in men and 45% to 52% in women, and it is estimated that in the United Kingdom, 41% to 48% of men and 35% to 43% of women will be obese [2]. Obesity is well known to be a risk factor for different medical conditions, leading to increased morbidity and mortality [2,3]. Various interacting factors influence the prevalence of obesity, including people’s upbringing, lifestyle, environment, and genetics [4]. Over the past decades, numerous strategies for losing weight have been developed that mainly focus on reducing calorie intake and increasing energy expenditure [1]. It is important to tackle obesity early on, as the ability of a person to increase his or her activity levels decreases as his or her weight increases (particularly BMI >40) [4].

The rapid development of technology has led to a growing market of wearable devices claiming to help people lose weight. Over 100 million wearable devices were sold in 2016, and sales were expected to continue to rise over the next years [5]. Wearable technology refers to any electronic device that is worn on the body, commonly being fitness trackers containing some form of an activity monitor.

In combination with an effective weight management intervention based on a behavior change model, wearable technologies can help people lose weight through various means, eg, by promoting physical exercise, by monitoring food consumption, or by encouraging interuser communication and support [6]. Research on the effectiveness of interventions delivered by wearable devices suggests that these interventions can help lose weight [7]. However, long-term weight loss (>1 year) is often unsuccessful [8]. Wearable devices have only demonstrated a statistically significant weight loss lasting for a few weeks, which greatly reduces the potential usefulness of these devices [7]. Digital wearables could be a novelty that wears off over time, rather than being part of a sustained lifestyle change [9].

Previous research on weight loss interventions without wearable technology has shown that over a 5-year period, only 20% of individuals maintained a weight loss of more than 5 kg (after an initial loss of around 10 kg) [10,11]. Therefore, this review focused on studies that can aid long-term weight loss. Evidence on the long-term effects of wearables to manage or prevent obesity could be relevant for people seeking to reach a healthy weight and for their medical practitioners [12].

This study systematically reviewed the use of wearable devices for long-term weight loss in overweight and obese adults. This review had four objectives: (1) to investigate the effects of using wearable devices on physical activity and weight outcomes, (2) to examine the duration of wearable technology use, (3) to assess the accuracy of wearable technology vs self-reporting, and (4) to explore the use of wearable technology by people with specific medical conditions.

## Methods

### Protocol

A protocol was registered with the International Prospective Register for Systematic Reviews (CRD42018096932), with the review structure following the Preferred Reporting Items for Systematic Reviews and Meta-Analyses (PRISMA) guidelines (Multimedia Appendix 1). We narrowed down the review question of the protocol, focusing on long-term weight management.

### Eligibility Criteria

Textbox 1 summarizes the inclusion and exclusion criteria for the participant, intervention, comparators, outcomes, and study types of this systematic review.

**Inclusion and exclusion criteria for study selection.**

- Population: We included studies with obese or overweight adult participants. *Overweight* was defined as having a BMI ranging between 25 kg/m2 and 29.99 kg/m2 or as defined by the study. *Obese* was defined as having a BMI of 30 kg/m2 or more. Participants in hospital settings were excluded as these studies were unlikely to focus on long-term effects and as they do not represent the real-world use of wearable devices.
- Intervention: Interventions included digital wearable technologies used for monitoring or managing weight. Studies that only included a mobile phone app were excluded.
- Comparators: Comparators included traditional behavioral weight loss approaches, usual care, or another intervention. Studies that did not have a comparator were also included if they met the other inclusion criteria.
- Outcomes: The primary outcome was change in physical activity and weight after using digital wearable technology for at least a year. Secondary outcomes were the duration of wearable technology use, the accuracy of wearable technology vs self-reporting, and the use of wearable technology by people with specific medical conditions.
- Study types: All types of studies were included. Owing to the rapid advances in technology, studies from only the past 10 years were used—from 2008 onward.

### Information Sources and Search

We searched the following databases: Medical Literature Analysis and Retrieval System Online, Compendex, ScienceDirect, Cochrane Central, Scopus, and EMBASE through Ovid. Multimedia Appendix 2 outlines the search terms. Data published before 2008 were not included as these data are not reflective of the rapid change in the use of mobile phones and wearables. Keywords related to participant, intervention, comparators, and outcome items were used to search for relevant papers. A librarian was consulted for advice on the searches. The search was adjusted and modified for each database.

### Study Selection

The references found were imported into EndNote X9 (Clarivate, Pennsylvania), and duplicates were removed. Overall, 2 reviewers conducted title and abstract screening. Any differences in the chosen studies were discussed until a consensus was reached. The full texts of potentially eligible studies were retrieved and analyzed for eligibility by 2 reviewers.

### Data Collection Process and Items

A standardized data extraction sheet was used to extract data. The extracted data included the title, the research question, the data sources, how the data were analyzed, the main findings, and the conclusions.

### Quality Appraisal of Individual Studies

All included studies underwent a methodological quality appraisal. Relevant appraisal tools were used for different study designs. The Cochrane risk of bias tool was used for randomized controlled trials (RCTs), Risk Of Bias In Nonrandomized Studies-of Interventions was used for nonrandomized studies of interventions, A MeaSurement Tool to Assess systematic Reviews was used for systematic reviews, and *6 questions to trigger critical thinking* were used for qualitative papers [13-16].

### Synthesis of Results

We have provided a narrative overview and tabular summary of the findings. A meta-analysis of the studies could not be conducted because of the heterogeneity in their interventions, participants, and outcomes.

## Results

### Study Selection and Characteristics

**Figure 1:**
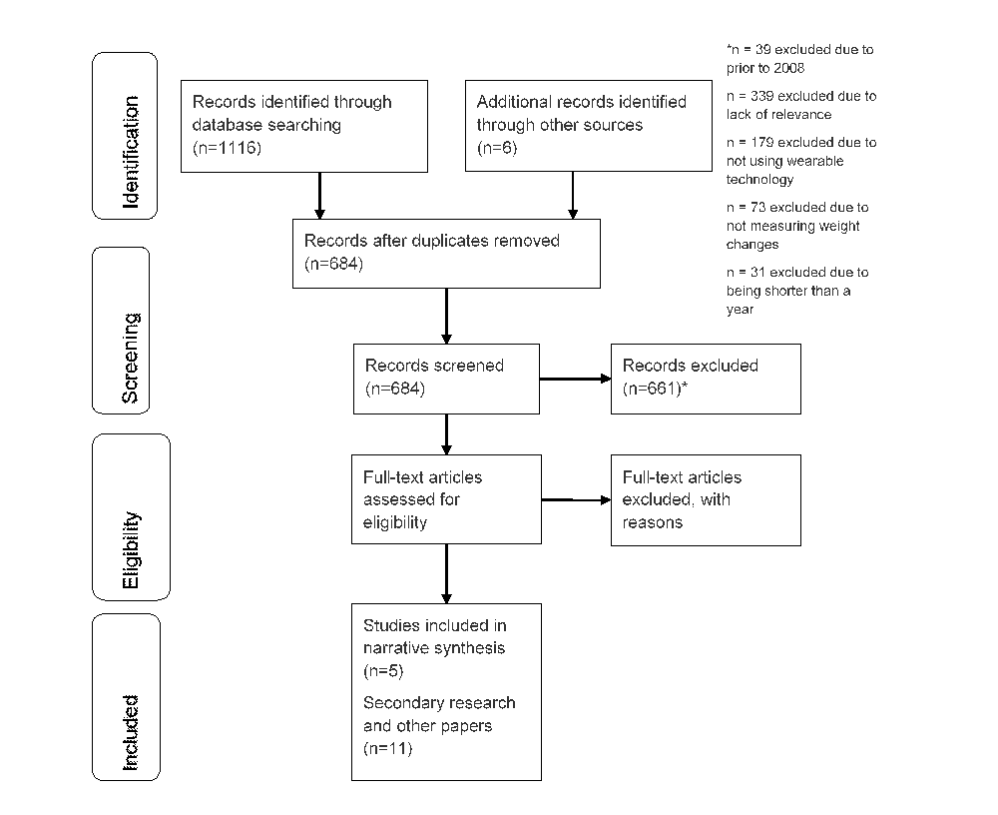
Preferred Reporting Items for Systematic Reviews and Meta-Analyses (PRISMA) flow diagram showing the selection of included studies.

We found 1116 references, and after removing duplicates and adding six references identified through searching reference lists of included studies, 684 titles and abstracts were screened (Figure 1). Furthermore, 44 full texts were assessed for inclusion, of which 28 were excluded (Multimedia Appendix 3). We included five intervention studies: four RCTs and one nonrandomized study of an intervention as shown in Table 1. In addition, we used insights from six systematic reviews, four commentary papers, and a dissertation for additional insights (Table 2).

**Table 1:** Included intervention studies.

Study	Author (year)	Title	Type of paper
1	Fazzino et al (2017) [17]	Change in physical activity during a weight management intervention for breast cancer survivors: Association with weight outcomes	Randomized controlled trial
2	Chiang et al (2017) [18]	Potential impact of wearable technology as part of a multidisciplinary treatment strategy for weight regain following RYGB^a^	Randomized controlled trial
3	Jakicic et al (2016) [19]	Effect of wearable technology combined with a lifestyle intervention on long-term weight loss: The IDEA randomized clinical trial	Randomized controlled trial
4	Jakicic et al (2012) [20]	Effect of a stepped-care intervention approach on weight loss in adults: A randomized clinical trial	Randomized controlled trial
5	Sepah et al (2015) [21]	Long-term outcomes of a Web-based diabetes prevention program: 2-year results of a single-arm longitudinal study	Nonrandomized study of an intervention

^a^RYGB: Roux-en-Y gastric bypass.

**Table 2:** Secondary research and other papers.

Study	Author (year)	Title	Type of paper
6	Podina and Fodor (2018) [16]	Critical review and meta-analysis of multicomponent behavioural e-health interventions for weight loss	Systematic review
7	Lyzwinski (2014) [22]	A systematic review and meta-analysis of mobile devices and weight loss with an intervention content analysis	Systematic review
8	Allen et al (2014) [23]	Technology-assisted weight management interventions: Systematic review of clinical trials	Systematic review
9	Kelders et al (2012) [24]	Persuasive system design does matter: A systematic review of adherence to Web-based interventions	Systematic review
10	Goode et al (2017) [25]	The impact of interventions that integrate accelerometers on physical activity and weight loss: A systematic review	Systematic review
11	Coons et al (2012) [26]	Technology interventions to curb obesity: A systematic review of the current literature	Systematic review
12	Kulick (2017) [27]	Wearable technology and long-term weight loss	Comment
13	Jakicic and Belle (2017) [28]	Wearable technology and long-term weight loss-Reply	Comment
14	Hekler et al (2016)	Advancing models and theories for digital behavior change interventions [6]	Comment
15	Dyer (2016) [29]	Wearable fitness device does not help maintain weight loss, study finds: Fitness device doesn’t maintain weight loss	Comment
16	Assar (2018) [30]	Evidence-based psychotherapeutic interventions and mHealth for weight management in overweight: A biopsychosocial framework	Dissertation

### Risk of Bias Within Studies

There was a large variation in bias in all the included papers (Multimedia Appendix 4). In most of the RCTs, there was a high risk of bias because of the lack of blinding of participants, as a blinded version of a wearable device intervention is not possible. All the included RCTs used random sequence generation to generate groups, although information about allocation concealment was missing in half of them.

### Synthesis of Results

Table 3 outlines the studies that contributed findings to the fulfillment of the objectives of this review: physical activity and weight outcomes, duration of wearable technology use, accuracy of wearable technology over self-reporting, and use by people with specific medical conditions.

**Table 3:** Included intervention studies and findings.

Study	Author (year)	Description	Physical activity and weight outcomes	Long-term use	Accuracy compared with self-reporting	Population with a specific medical condition
1	Fazzino et al (2017) [17]	An RCT^a^ assessing the effects of mobile health weight management on physical activity, weight loss, and weight maintenance	Yes	Yes	Yes	Breast cancer prevention
2	Chiang et al (2017) [18]	An RCT on the weight loss after repaired RYGB^b^ surgery, with and without wearable devices	Yes	No	No	After repair of failed Roux-en-Y gastric bypass
3	Jakicic et al (2016) [19]	An RCT comparing outcomes of technology-enhanced interventions with standard behavioral interventions	Yes	Yes	Yes	No
4	Jakicic et al (2012) [20]	An RCT comparing a standard and stepped-care intervention in weight loss	No	Yes	No	No
5	Sepah et al (2015) [21]	A diabetes prevention study measuring the outcomes of weight and hemoglobin A_1c_	No	Yes	No	Prediabetes

^a^RCT: randomized controlled trial.

^b^RYGB: Roux-en-Y gastric bypass.

### Association Between Weight Outcomes and Change in Physical Activity

The three studies reporting on weight loss and physical activity outcomes concluded that using wearable devices had a benefit on these outcomes, but not compared with the comparator groups. Study 1 showed a significant rise in moderate-to-vigorous physical activity over 18 months and divided participants into high or low original weight loss and high or low weight regain groups. At 6 months, the high weight loss groups had significantly higher level of moderate-to-vigorous physical activity than the low weight loss group. However, at 12 and 18 months, the high loss and high regain groups’ level of moderate-to-vigorous physical activity fell, leaving the high loss and low regain group with a significantly higher level of moderate-to-vigorous physical activity than all other groups.

Study 2 compared 27 individuals using wearable devices with 260 individuals who were not using a wearable device. A total of 8000 steps per day was recommended for the intervention group, but it is not noted whether the comparator group were given similar recommendations. A significant benefit was only found at 2 years (*P*=.03).

In both the standard and wearable device groups of study 3, there was an increase in the duration of moderate-to-vigorous physical activity sessions ≥10 min over a 12-month period, but there was no statistically significant difference between the two groups. The percentage of weight lost did differ, with significantly greater weight loss in the comparator group compared with the wearable devices group from 12 months onward. Study 3 did not find an association between physical activity and weight loss within its groups.

### Maintenance of Wearable Technology Use

Retention was fairly high in four studies, but study 2 did not provide data (Table 4). Study 4 was the only study mentioning to offer a monetary incentive for assessments (US $10-$25). Study 4 compared a standard intervention with a stepped-care intervention, where the intensity of support (such as telephone intervention and additional individual sessions) increased if certain goals were not met.

**Table 4:** Retention rate across included intervention studies.

Study	Author (year)	Description	Retention at 6 months, %	Retention at 18 months, %	Retention at 24 months, %	Notes
1	Fazzino et al (2017) [17]	An RCT^a^ assessing the effects of mobile health weight management on physical activity, weight loss, and weight maintenance	N/A^b^	68	N/A	80% maintained intervention use at 18 months but without valid accelerometer data.
2	Chiang et al (2017) [18]	An RCT on the weight loss after repaired RYGB^c^ surgery, with and without wearable devices	N/A	N/A	N/A	N/A
3	Jakicic et al (2016 [19]	An RCT comparing outcomes of technology-enhanced interventions with standard behavioral interventions	N/A	N/A	75	N/A
4	Jakicic et al (2012) [20]	An RCT comparing a standard and stepped-care intervention in weight loss	N/A	72	N/A	N/A
5	Sepah et al (2015) [21]	A diabetes prevention study measuring the outcomes of weight and hemoglobin A_1c_	79.1	N/A	70.1	N/A

^a^RCT: randomized controlled trial.

^b^N/A: not applicable.

^c^RYGB: Roux-en-Y gastric bypass.

### Accuracy of Wearable Technology Versus Self-Reporting

Accuracy was reported by two studies. Study 1 found that self-reported moderate-to-vigorous physical activity was significantly higher than that recorded by the wearable device. The groups with poorer outcomes (low loss or high regain) had larger discrepancies between the two methods. The self-reported and accelerometer-derived moderate-to-vigorous physical activities were most similar to the high loss and low regain group. the moderate-to-vigorous physical activity data from self-reporting and the accelerometer were, however, collected on different weeks. However, the high loss and low regain group still overestimated moderate-to-vigorous physical activity, and the overestimation did not reduce over time. Study 1 suggested “social desirability to report physical activity adherence,” with participants inflating self-reported moderate-to-vigorous physical activity.

### Use of Wearable Technology by People With Specific Medical Conditions

Overall, three out of the five studies focused on populations with a specific medical condition (Table 4). This included a history of breast cancer (study 1), repair of failed Roux-en-Y gastric bypass (study 2), or prediabetes (study 5). Study 1 analyzed those who attended the visits but had invalid or missing data and found no significant difference in *cancer treatment–related variables*. Study 5 measured blood glucose (hemoglobin A_1c_) levels and showed a significant beneficial reduction over 24 months.

## Discussion

### Principal Findings

This review showed some evidence that wearable devices can improve long-term physical activity and weight loss outcomes, but there was not enough evidence to show a benefit over the comparator methods. The comparator interventions differed among studies, which adds to the difficultly in determining the impact on outcomes. Although the term *standard* was used, there was no standardization in the comparators’ intervention, with different levels of support and procedures.

Overall physical activity levels increased from baseline, but there was no difference between wearable and comparator interventions. Study 1 found that those who sustained higher physical activity levels were more likely to maintain weight loss. Retention was fairly high in the included intervention studies. The mechanism through which wearable devices have an effect compared with other methods was not known as diet and physical activity were not different. The accuracy of wearable devices varied, which could be explained by the different features and technology of wearables. A total of three included studies focused on populations with a specific medical condition. The difference in populations added a challenge to comparing the studies as the results of a study on the weight management of patients with one medical condition may not apply to patients with another medical condition or the general population.

### Limitations

There were only five studies with a relatively small sample size assessing the long-term use of wearable devices. It was not possible to undertake a meta-analysis because of the heterogeneity among participants, wearables, methods, and outcomes. The included studies were likely to suffer from bias. Wearable device interventions cannot be *blinded* to the user. Only outcome assessors could have been blinded, which most studies did not attempt to do. The use of wearables in these studies may not be applicable to real-world scenarios as the companies selling these wearable devices do not offer the support that was offered by researchers in the studies. A limitation of this review is that we only conducted a basic search limited to a few keywords and phrases. In addition, databases such as Institute of Electrical and Electronics Engineers Xplore and Cumulative Index of Nursing and Allied Health Literature for clinical and behavioral science research were not searched.

### Comparison With Prior Work

It is important to retain participants in studies to separate the effect of the study design and intervention [31]. Study 5 compared a standard intervention with a stepped-care intervention where the intensity of support (such as telephone intervention and additional individual sessions) increased if certain goals were not met. Interventions of this kind have been shown to reduce attrition [32]. Other strategies for improving long-term data collection are offering incentives, reducing barriers by offering *alternative data collection modes*, and reminder calls [32]. Improving adoption and retention through methods such as monetary incentives could be counterproductive as this is not possible in real-life settings.

Consciously or subconsciously, self-reported physical activity levels are often overestimated [33]. Wearable devices are more accurate at estimating physical activity levels than self-reporting, though a truly objective method is currently not available for everyday purposes. Accelerometers, which were used in the wearable devices in the included studies, can lead to different estimates, even when using the same device [34]. The accuracy of heart rate monitors has been reported to be higher but still insufficient [35].

Wearable devices have shown benefit in managing medical conditions, eg, diabetes [36]. Studies with populations having medical conditions or risk factors could suffer from higher dropout because of the higher risk of a medical event. However, having a specific condition or medical event could be a stronger motivation than having a *vaguer* risk factor such as being overweight or obese [37].

### Recommendations for Future Work

Different aspects of weight loss maintenance and wearable devices have been studied, but large areas are still unknown. These include the mechanisms through which using wearable devices can lead to weight loss and studies into the usefulness of wearable devices for long-term weight management.

Those who managed to sustain raised physical activity levels had a weight maintenance benefit. Not all groups managed to sustain increased activity levels, and it would be valuable to understand why. Individuals who sustained exercise could have been more likely to commit to other lifestyle changes around weight management.

Investigating the reasons for dropout could help to understand to what extent this is caused by study design and/or flaws in wearable devices. Discovering how wearable devices are being used, and whether their use is improved through outside support, would give valuable information for designing more effective wearable devices. It could also help health care practitioners to advice and support people who are trying to lose weight and are interested in using wearable devices.

### Conclusions

We found a small number of long-term studies showing some evidence that wearable devices can improve long-term physical activity and weight loss outcomes, but there was not enough evidence to show a benefit over the comparator methods. A major issue is the challenge to separate the effect of the decreasing use of wearable devices over time from the effect of wearable devices on the outcomes. Consistency in study methods is needed in future long-term studies on the use of wearable devices for weight loss.

## Appendix

Multimedia Appendix 1 Identify the report as a systematic review, meta-analysis, or both.

Multimedia Appendix 2 Results found in each database and the search strings used.

Multimedia Appendix 3 Excluded studies.

Multimedia Appendix 4 Quality appraisal of randomized controlled trials.

## Abbreviations

PRISMA: Preferred Reporting Items for Systematic Reviews and Meta-Analyses
RCT: randomized controlled trial
