# Supplementary material for: Long-Term Weight Management Using Wearable Technology in Overweight and Obese Adults: Systematic Review
Source: JMIR Mhealth Uhealth. 2020 Mar 10;8(3):e13461. doi: 10.2196/13461 (PMC7093773; doi:10.2196/13461)
Supplement: Multimedia Appendix 2 [file mhealth_v8i3e13461_app2.docx]

# Appendix B

Results found in each database and the search strings used

| Database | Search Strings | Results |
| --- | --- | --- |
| PubMed | (Obesity OR Obese OR Overweight OR BMI) AND (Wearable* OR “mobile health” OR mhealth) AND (“Long Term” OR “Follow Up” OR "weight maintenance" OR "weight loss maintenance" OR “weight management”) NOT child* NOT Paediatric* | 170 |
| Compendex ScienceDirect | NOT child (Obesity OR Obese OR Overweight OR BMI AND wearable* OR “mobile health” OR mhealth AND "Long Term" OR "Follow Up" OR "weight maintenance" OR "weight loss maintenance" OR “weight management”) | 57 |
| Cochrane Central | (Obesity OR Obese OR Overweight OR BMI) AND (Wearable* OR “mobile health” OR mhealth) AND (“Long Term” OR “Follow Up” OR “weight loss maintenance” OR “weight maintenance” OR “weight management”) NOT child* NOT Paediatric* | 17 |
| Scopus | ( ALL ( obesity OR obese OR overweight OR BMI ) AND ALL ( wearable* OR "mobile health" OR mhealth ) AND ALL ( "Long Term" OR "Follow Up" OR "weight loss maintenance" OR "weight maintenance" OR "weight management" ) AND NOT ALL ( child* ) AND NOT ALL ( paediatrics* ) ) | 88 |
| Embase | (Obesity OR Obese OR Overweight OR BMI) AND (Wearable* OR mobile health OR mhealth) AND (Long Term OR Follow Up OR weight maintenance OR weight loss maintenance OR weight management) NOT child* NOT Paediatric* | 784 |
| Total number |  | 1116 |
