# Supplementary material for: Long-Term Weight Management Using Wearable Technology in Overweight and Obese Adults: Systematic Review
Source: JMIR Mhealth Uhealth. 2020 Mar 10;8(3):e13461. doi: 10.2196/13461 (PMC7093773; doi:10.2196/13461)
Supplement: Multimedia Appendix 3 [file mhealth_v8i3e13461_app3.docx]

# Appendix C Excluded studies

1. Coorey G, Neubeck L, Mulley J et al. Effectiveness, acceptability and usefulness of mobile applications for cardiovascular disease self-management: Systematic review with meta-synthesis of quantitative and qualitative data. European Journal of Preventive Cardiology 2018;25:505-521. doi:10.1177/2047487317750913

Further reading into the studies included showed that the ten studies included had no studies over a year long.

1. Does Technology Accelerate Weight Loss? Intern med alert 2016;38(22):175.

Turned out to be a duplicate of ‘Effect of wearable technology combined with a lifestyle intervention on long-term weight loss: The IDEA randomized clinical trial’, so removed.)

1. Kramer-Jackman K, Popkess-Vawter S. Method for Technology-Delivered Healthcare Measures. CIN: Computers, Informatics, Nursing 2011;29:730-740. doi:10.1097/ncn.0b013e318224b581y

Not relevant

1. Waterlander W, Whittaker R, McRobbie H et al. Development of an Evidence-Based mHealth Weight Management Program Using a Formative Research Process. JMIR mhealth and uhealth 2014;2:e18. doi:10.2196/mhealth.2850

Focus on development, not relevant

1. Bardus M, van Beurden S, Smith J et al. A review and content analysis of engagement, functionality, aesthetics, information quality, and change techniques in the most popular commercial apps for weight management. International Journal of Behavioral Nutrition and Physical Activity 2016;13. doi:10.1186/s12966-016-0359-9

Not wearable technology

1. Spring B, Duncan J, Janke E et al. Integrating Technology Into Standard Weight Loss Treatment. JAMA Internal Medicine 2013;173:105. doi:10.1001/jamainternmed.2013.1221

Not wearable technology

1. Godino J, Merchant G, Norman G et al. Using social and mobile tools for weight loss in overweight and obese young adults (Project SMART): a 2 year, parallel-group, randomised, controlled trial. The Lancet Diabetes & Endocrinology 2016;4:747-755. doi:10.1016/s2213-8587(16)30105-x

Not wearable technology

1. Haapala I, Barengo N, Biggs S et al. Weight loss by mobile phone: a 1-year effectiveness study. Public Health Nutrition 2009;12:2382. doi:10.1017/s1368980009005230

Not wearable technology

1. Shapiro J, Koro T, Doran N et al. Text4Diet: A randomized controlled study using text messaging for weight loss behaviors. Preventive Medicine 2012;55:412-417. doi:10.1016/j.ypmed.2012.08.011

Not wearable technology

1. Hutchesson M, Rollo M, Krukowski R et al. eHealth interventions for the prevention and treatment of overweight and obesity in adults: a systematic review with meta-analysis. Obesity Reviews 2015;16:376-392. doi:10.1111/obr.12268

Not wearable technology

1. Raaijmakers L, Pouwels S, Berghuis K et al. Technology-based interventions in the treatment of overweight and obesity: A systematic review. Appetite 2015;95:138-151. doi:10.1016/j.appet.2015.07.008

No new included studies in the systematic review

1. Webb T, Joseph J, Yardley L et al. Using the Internet to Promote Health Behavior Change: A Systematic Review and Meta-analysis of the Impact of Theoretical Basis, Use of Behavior Change Techniques, and Mode of Delivery on Efficacy. Journal of Medical Internet Research 2010;12:e4. doi:10.2196/jmir.1376

Searched databases before 2008

1. Riley W, Rivera D, Atienza A et al. Health behavior models in the age of mobile interventions: are our theories up to the task?. Translational Behavioral Medicine 2011;1:53-71. doi:10.1007/s13142-011-0021-7

Not long term

1. Biddle S, Edwardson C, Wilmot E et al. A Randomised Controlled Trial to Reduce Sedentary Time in Young Adults at Risk of Type 2 Diabetes Mellitus: Project STAND (Sedentary Time ANd Diabetes). PLOS ONE 2015;10:e0143398. doi:10.1371/journal.pone.0143398

Did not record weight loss

1. Shuger S, Barry V, Sui X et al. Electronic feedback in a diet- and physical activity-based lifestyle intervention for weight loss: a randomized controlled trial. International Journal of Behavioral Nutrition and Physical Activity 2011;8:41. doi:10.1186/1479-5868-8-41

Only 9 months long, so not included

1. Pellegrini C, Verba S, Otto A et al. The Comparison of a Technology-Based System and an In-Person Behavioral Weight Loss Intervention. Obesity 2011;20:356-363. doi:10.1038/oby.2011.13

Only 6 months long, so not included

1. Bardus M, Smith J, Abraham C. Are eHealth interventions for obesity prevention effective? A systematic review of reviews. European Journal of Public Health 2015;25. doi:10.1093/eurpub/ckv172.081

No new included studies in the systematic review

1. Luley C, Blaik A, Götz A et al. Weight Loss by Telemonitoring of Nutrition and Physical Activity in Patients with Metabolic Syndrome for 1 Year. Journal of the American College of Nutrition 2014;33:363-374. doi:10.1080/07315724.2013.875437

Only 6 months long, so not included

1. Runkle D. The mHealth revolution. SciTech Lawyer. 2013;9(3)4(9):24–25, 30–31.

No longer available

1. Hekler EB, Buman MP, Poothakandiyil N, et al. Exploring behavioral markers of long-term physical activity maintenance: a case study of system identification modeling within a behavioral intervention. Health education & behavior : the official publication of the Society for Public Health Education 2013;40(1 Suppl):51s-62s.

Not wearable technology

1. Morgan PJ, Lubans DR, Collins CE, et al. 12-month outcomes and process evaluation of the SHED-IT RCT: an internet-based weight loss program targeting men. Obesity (Silver Spring, Md) 2011;19(1):142-51.

Not wearable technology

1. Coffeng J, van der Ploeg H, Castellano J, et al. A 30-month worksite-based lifestyle program to promote cardiovascular health in middle-aged bank employees: Design of the TANSNIP-PESA randomized controlled trial. Am Heart J 2017;184:121-32.

This was excluded after a full text review showed that it was simply about the development of the trial, rather than the trial itself. The trial itself would have been included, but the results have not yet come in regarding it. Without any data, it was not included.

1. Foley P, Steinberg D, Levine E, et al. Track: A randomized controlled trial of a digital health obesity treatment intervention for medically vulnerable primary care patients. Contemporary clinical trials 2016;48:12-20.

As with the previous excluded study in the full text assessment, this was excluded as it was the planning, and preliminary information on populations, of a study which does not yet have results in for it. Like before, the study itself would have fitted the inclusion criteria.

1. Guertler D, Vandelanotte C, Kirwan M, et al. Engagement and nonusage attrition with a free physical activity promotion program: The case of 10,000 steps Australia. Journal of medical Internet research 2015;17(7):No-Specified.

This study was excluded as a full text read through showed that the time period of most information gathered in the study was under a year in length, meaning it would not provide evidence for long term impacts of wearable technology on health and weight.

1. Kim JY, Wineinger NE, Taitel M, et al. Self-monitoring utilization patterns among individuals in an incentivized program for healthy behaviors. Journal of medical Internet research 2016;18(11):83-97.

This study also was shown to have a time period of study of less than a year between intervention and then the latest follow up measurements, so it was excluded as it did not meet the criteria of showing maintenance (which was defined as a period of at least a year).

1. Raaijmakers LCH, Pouwels S, Berghuis KA, et al. Technology-based interventions in the treatment of overweight and obesity: A systematic review. Appetite 2015;95:138-51.

The referenced studies of this systematic review were looked through, and none of the ones analysed fit the inclusion criteria completely, due to too short time span, or not focus on wearable technologies, or something else. Due to the lack of relevant studies, this systematic review was also excluded.

1. Stephenson A, McDonough SM, Murphy MH, et al. Using computer, mobile and wearable technology enhanced interventions to reduce sedentary behaviour: A systematic review and meta-analysis. The international journal of behavioral nutrition and physical activity 2017;14.

As with the previous exclusion, all of the referenced papers of this systematic review did not fit the inclusion criteria, so the systematic review was excluded due to lack of relevancy to the research question.

1. Urrea B, Kianoush S, Feldman DI, et al. Long-term impact of the mActive physical activity trial on cardiometabolic health. European Heart Journal 2016;37(Supplement 1):335.

Excluded due to too short of a time period (5 weeks).
