# Supplementary material for: Long-Term Weight Management Using Wearable Technology in Overweight and Obese Adults: Systematic Review
Source: JMIR Mhealth Uhealth. 2020 Mar 10;8(3):e13461. doi: 10.2196/13461 (PMC7093773; doi:10.2196/13461)
Supplement: Multimedia Appendix 4 [file mhealth_v8i3e13461_app4.docx]

# Appendix D

Quality appraisal of randomized controlled trials

| Study - RCT | Random Sequence Generation | Allocation Concealment | Blinding | Incomplete outcome data | Selective Reporting | Other |
| --- | --- | --- | --- | --- | --- | --- |
| Change in physical activity during a weight management intervention for breast cancer survivors: Association with weight outcomes | + | ? | +++ | + | + | + |
| Potential impact of wearable technology as part of a multidisciplinary treatment strategy for weight regain following roux-en-y gastric bypass | + | ? | +++ | +++ | +++! | +++ |
| Effect of wearable technology combined with a lifestyle intervention on long-term weight loss: The IDEA randomized clinical trial | + | + | +++ | + | + | + |
| Effect of a stepped-care intervention approach on weight loss in adults: a randomized clinical trial | + | + | +++ | +++ | + | + |

Quality appraisal of non-randomised study

| Study – NRSI | Confounding | Selection | Bias in Classification | Deviations of interventions | Missing Data | Measurement of Outcomes | Selection of Reported Results | Total Bias |
| --- | --- | --- | --- | --- | --- | --- | --- | --- |
| Long-term outcomes of a Web-based diabetes prevention program: 2-year results of a single-arm longitudinal study | ++ | ++ | ++ | ++ | ++ | ++ | + | ++ |

| Low Risk | + |
| --- | --- |
| **Moderate Risk** | ++ |
| **Serious Risk** | +++ |
| **Critical Risk** | ++++ |
| **Unknown Risk/No Data** | ? |

### Quality appraisal of systematic reviews

|  |  | Critical review and meta-analysis of multicomponent behavioural e-health interventions for weight loss | The Impact of Interventions that Integrate Accelerometers on Physical Activity and Weight Loss: A Systematic Review | A systematic review and meta-analysis of mobile devices and weight loss with an intervention content analysis | Technology-assisted weight management interventions: systematic review of clinical trials | Persuasive system design does matter: a systematic review of adherence to web-based interventions | Technology Interventions to Curb Obesity: A Systematic Review of the Current Literature |
| --- | --- | --- | --- | --- | --- | --- | --- |
| **1** | **Did the research questions and inclusion criteria for the review include the components of PICO?** | YES | YES | YES | YES | NO | YES |
| For Yes: | Population | YES | YES | YES | YES |  | YES |
|  | Intervention | YES | YES | YES | YES | YES | YES |
|  | Comparator group | YES | YES | YES | YES |  | YES |
|  | Outcome | YES | YES | YES | YES | YES | YES |
| Optional: | Timeframe for follow up |  | YES |  |  |  |  |
| **2** | **Did the report of the review contain an explicit statement that the review methods were established prior to the conduct of the review and did the report justify any significant deviations from the protocol?** | NO | NO | PARTIAL YES |  | NO | NO |
| For Partial Yes: The authors state that they had a written protocol or guide that included ALL the following: | Review question(s) |  |  | YES |  | YES |  |
|  | A search strategy |  | YES | YES | YES | YES | YES |
|  | Inclusion/exclusion criteria |  | YES | YES |  |  |  |
|  | A risk of bias assessment |  |  |  |  |  |  |
| For Yes: As for partial yes, plus the protocol should be registered, and should also have specified ALL: | A meta-analysis/synthesis plan, if appropriate |  |  |  |  |  |  |
|  | A plan for investigating causes of heterogeneity |  |  |  |  |  |  |
|  | Justification for any deviations from the protocol |  |  |  |  |  |  |
| **3** | **Did the review authors explain their selection of the study designs for inclusion in the review?** | NO | NO | NO | NO | NO | NO |
| For Yes: Either ONE of the following: | Explanation for including only RCTs |  |  |  |  |  |  |
|  | Explanation for including only NRSI |  |  |  |  |  |  |
|  | Explanation for including both RCTs and NRSI |  |  |  |  |  |  |
| **4** | **Did the review authors use a comprehensive literature search strategy?** | PARTIAL YES | PARTIAL YES | PARTIAL YES | PARTIAL YES | PARTIAL YES | PARTIAL YES |
| For Partial Yes: | Searched at least 2 databases (relevant to research question) | YES | YES | YES | YES | YES | YES |
|  | Provided key word and/or search strategy | YES | YES | YES | YES | YES | YES |
|  | Justified publication restrictions (e.g. Language) | YES | YES | YES | YES | YES | YES |
| For Yes: | Searched the reference lists / bibliographies of included studies |  | YES | YES |  |  |  |
|  | Searched trial/study registries | YES |  |  |  |  |  |
|  | Included/consulted content experts in the field |  |  |  |  |  |  |
|  | Where relevant, searched for grey literature | YES |  | YES |  |  |  |
|  | Conducted search within 24 months of completion of the review | YES |  |  |  |  |  |
| **5** | **Did the review authors perform study selection in duplicate?** | YES | YES | NO | YES | YES | YES |
| For Yes: Either ONE of the following: | At least two reviewers independently agreed on selection of eligible studies and achieved consensus on which studies to include | YES | YES |  |  | YES |  |
|  | Two reviewers selected a sample of eligible studies and achieved good agreement (at least 80 percent), with the remainder selected by one reviewer |  |  |  | YES |  | YES |
| **6** | **Did the review authors perform data extraction in duplicate?** | YES | YES | NO | NO |  | NO |
| For Yes: Either ONE of the following: | At least two reviewers achieved consensus on which data to extract from included studies | YES | YES |  |  |  |  |
|  | Two reviewers extracted data from a sample of eligible studies and achieved good agreement (at least 80 percent), with the remainder extracted by one reviewer |  |  |  |  |  |  |
| **7** | **Did the review authors provide a list of excluded studies and justify the exclusions?** | PARTIAL YES | NO | YES | YES | PARTIAL YES | PARTIAL YES |
| For Partial Yes: | Provided a list of all potentially relevant studies that were read in full-text form but excluded from the review | YES |  | YES | YES | YES | YES |
| For Yes: | Justified the exclusion from the review of each potentially relevant study |  |  | YES | YES |  |  |
| **8** | **Did the review authors describe the included studies in adequate detail?** | PARTIAL YES | PARTIAL YES | YES | PARTIAL YES | NO | YES |
| For Partial Yes: | Described populations | YES | YES | YES | YES |  | YES |
|  | Described interventions | YES | YES | YES | YES | YES | YES |
|  | Described comparators | YES | YES | YES | YES |  | YES |
|  | Described outcomes | YES | YES | YES | YES |  | YES |
|  | Described research designs | YES | YES | YES | YES |  | YES |
| For Yes: | Described population in detail |  |  | YES | YES |  | YES |
|  | Described intervention in detail (including doses where relevant) |  | YES | YES | YES |  | YES |
|  | Described comparator in detail (including doses where relevant) |  | YES | YES |  |  | YES |
|  | Described study’s setting |  |  | YES |  |  | YES |
|  | Timeframe for follow-up | YES | YES | YES | YES | YES | YES |
| **9** | **Did the review authors use a satisfactory technique for assessing the risk of bias (rob) in individual studies that were included in the review?** | YES | YES | YES | YES | NO | NO |
| For Partial Yes: RCT must also have assessed RoB: | Unconcealed allocation | YES | YES | YES | YES |  |  |
|  | Lack of blinding of patients and assessors when assessing outcomes (unnecessary for objective outcomes such as all-cause mortality) | YES | YES | YES | YES |  |  |
| For Yes: RCT must also have assessed RoB: | Allocation sequence that was not truly random | YES | YES | YES | YES |  |  |
|  | Selection of the reported result from among multiple measurements or analyses of a specified outcome | YES | YES | YES | YES |  |  |
| For Partial Yes: NRSI must also have assessed RoB: | From confounding |  |  |  |  |  |  |
|  | From selection bias |  |  |  |  |  |  |
| For Yes: NRSI must also have assessed RoB: | Methods used to ascertain exposures and outcomes |  |  |  |  |  |  |
|  | Selection of the reported result from among multiple measurements or analyses of a specified outcome |  |  |  |  |  |  |
| **10** | **Did the review authors report on the sources of funding for the studies included in the review?** | NO | NO | NO | NO | NO | NO |
| For Yes: | Must have reported on the sources of funding for individual studies included in the review |  |  |  |  |  |  |
| **11** | **If meta-analysis was performed did the review authors use appropriate methods for statistical combination of results?** | YES | YES | YES | N/A | N/A | N/A |
| For Yes: RCT: | The authors justified combining the data in a meta-analysis | YES | YES | YES |  |  |  |
|  | They used an appropriate weighted technique to combine study results and adjusted for heterogeneity if present | YES | YES | YES |  |  |  |
|  | Investigated the causes of any heterogeneity | YES | YES | YES |  |  |  |
| For Yes: NRSI: | The authors justified combining the data in a meta-analysis |  |  |  |  |  |  |
|  | They used an appropriate weighted technique to combine study results, adjusting for heterogeneity if present |  |  |  |  |  |  |
|  | They statistically combined effect estimates from NRSI that were adjusted for confounding, rather than combining raw data, or justified combining raw data when adjusted effect estimates were not available |  |  |  |  |  |  |
|  | They reported separate summary estimates for RCTs and NRSI separately when both were included in the review |  |  |  |  |  |  |
| **12** | **If meta-analysis was performed, did the review authors assess the potential impact of rob in individual studies on the results of the meta-analysis or other evidence synthesis?** | NO | YES | YES | N/A | N/A | N/A |
| For Yes: Either ONE of the following: | Included only low risk of bias RCTs |  |  |  |  |  |  |
|  | If the pooled estimate was based on RCTs and/or NRSI at variable rob, the authors performed analyses to investigate possible impact of rob on summary estimates of effect |  | YES | YES |  |  |  |
| **13** | **Did the review authors account for rob in individual studies when interpreting/ discussing the results of the review?** | YES | YES | YES | YES | NO | NO |
| For Yes: Either ONE of the following: | Included only low risk of bias RCTs |  |  | YES | YES |  |  |
|  | If RCTs with moderate or high rob, or NRSI were included the review provided a discussion of the likely impact of rob on the results | YES | YES |  |  |  |  |
| **14** | **Did the review authors provide a satisfactory explanation for, and discussion of, any heterogeneity observed in the results of the review?** | NO | YES | NO | NO | NO | YES |
| For Yes: Either ONE of the following: | There was no significant heterogeneity in the results |  |  |  |  |  |  |
|  | If heterogeneity was present the authors performed an investigation of sources of any heterogeneity in the results and discussed the impact of this on the results of the review |  | YES |  |  |  | YES |
| **15** | **If they performed quantitative synthesis did the review authors carry out an adequate investigation of publication bias (small study bias) and discuss its likely impact on the results of the review?** | YES | YES | YES | N/A | N/A | N/A |
| For Yes: | Performed graphical or statistical tests for publication bias and discussed the likelihood and magnitude of impact of publication bias | YES | YES | YES |  |  |  |
| **16** | **Did the review authors report any potential sources of conflict of interest, including any funding they received for conducting the review?** | YES | YES | YES | YES | YES | YES |
| For Yes: Either ONE of the following: | The authors reported no competing interests | YES | YES | YES | YES | YES | YES |
|  | The authors described their funding sources and how they managed potential conflicts of interest |  | | | | | |

### Quality appraisal of other papers

| Qualitative Piece | Where does this information come from? | What is being said? | How did they write this? | Who is telling me this? | When was this written? | Why has this been written? |
| --- | --- | --- | --- | --- | --- | --- |
| Evidence-based psychotherapeutic interventions and mhealth for weight management in overweight | A dissertation from a (successful) doctorate candidate. | A in depth and wide ranging examination of evidence based interventions for weight management. | A systematic review, along with further explorations into the implications. It is in depth and well-reasoned. | A doctorate candidate for psychology. | 2017 | To partially fulfil the requirements for a doctorate in psychology. |
| Wearable Technology and Long-term Weight Loss | The Journal of the American Medical Association (JAMA), a peer-review medical journal. | A summary of the results of the IDEA RCT, included in this systematic review | It is a concisely written summary of the key points from the RCT. | Written in to the editor from two medically affiliated doctors. | 2017 | To discuss the findings of the IDEA RCT |
| Wearable Technology and Long-term Weight Loss-Reply | The Journal of the American Medical Association, a peer-review medical journal. | A response to the previous piece, providing more details regarding the procedures used and efforts to reduce bias. | It is a well written response with more details regarding the RCT. | The original writers of the IDEA RCT | 2017 | As a response to the previous qualitative piece. |
| Advancing models and theories for digital behavior change interventions | The American Journal of Preventive Medicine, via PubMed Central | Recommendations for a framework for ‘digital behavior change interventions’ | It is a well written paper. | Several authors listed | 2016 | To improve strategies for modelling and theorising regarding ‘digital behavior change interventions’. |
| Wearable fitness device does not help maintain weight loss, study finds: Fitness device doesn't maintain weight loss, ^[24]^. | The BMJ, a peer reviews medical journal. | A summary of the IDEA RCT, ^[23]^. | It is a concisely written summary of the key points from the RCT. | A freelance journalist. | 2016 | To discuss the findings of the IDEA RCT, ^[23]^. |
